# Use of common spatial patterns for early detection of Parkinson’s disease

**DOI:** 10.1038/s41598-022-23247-0

**Published:** 2022-11-05

**Authors:** Aleš Smrdel

**Affiliations:** Faculty of Computer and Information Science, University of Ljubljana, Ljubljana, Slovenia

**Keywords:** Parkinson's disease, Parkinson's disease, Computer science, Parkinson's disease

## Abstract

One of the most common diseases that affects human brain is Parkinson’s disease. Detection of Parkinson’s disease (PD) poses a serious challenge. Robust methods for feature extraction allowing separation between the electroencephalograms (EEG) of healthy subjects and PD patients are required. We used the EEG records of healthy subjects and PD patients which were subject to auditory tasks. We used the common spatial patterns (CSP) and Laplacian mask as methods to allow robust selection and extraction of features. We used the derived CSP whitening matrix to determine those channels that are the most promising in the terms of differentiating between EEGs of healthy controls and of PD patients. Using the selection of features calculated using the CSP we managed to obtain the classification accuracy of 85% when classifying EEG records belonging to groups of controls or PD patients. Using the features calculated using the Laplacian operator we obtained the classification accuracy of 90%. Diagnosing the PD in early stages using EEG is possible. The CSP proved to be a promising technique to detect informative channels and to separate between the groups. Use of the combination of features calculated using the Laplacian offers good separability between the two groups.

## Introduction

Parkinson’s disease (PD) is one of the most common neurodegenerative diseases worldwide^1–3^. The incidence and prevalence of the disease have been rising rapidly in the last two decades^4–6^, although there are reports regarding decreasing trends in the PD in several countries world-wide^7^. One recent study^8^ also indicated, that the incidence and prevalence of the PD are slightly higher in the west, compared to the east. Manifestation of the PD differs among individuals, but cardinal signs of the PD are related to motor dysfunction, such as hypokinesia, resting tremors, problems with starting or also stopping the movement, lead pipe rigidity, and mask face and postural reflex impairment^9^. In addition to motor symptoms also non-motor symptoms, such as depression, anxiety, psychosis, apathy and/or impulse control disorders manifest in PD patients^10,11^. The manifestations of the PD change with passing of time, but the progress is related to the age of the patient rather than the age of the onset of the disease^12^.

The exact cause of the PD is not known, but studies suggest that different factors such as genetic and environmental might be responsible for the appearance^13^. No cure for the PD exists but different treatments can alleviate the symptoms and delay progression of the disease. Therefore it is important to diagnose the disease as early as possible. Clinical examinations, especially in early stages of the PD, are not very accurate^14^. The diagnosis of the PD is predominately based on motor symptoms, which develop after the neuropathological changes are already rampant^15^. Consequently, additional approaches are required, which could help diagnose the PD already in the early stages. In the past various approaches to diagnose the PD have been investigated and proposed.

Different non-invasive methods to characterize or detect the PD have been already proposed using e.g. voice measurements, gait or non-invasive signal acquisition techniques using wearable sensors. Several studies^16–19^ analyzed voice measurements from healthy subjects and PD patients to differentiate between the two groups (controls and PD patients). One study^20^ used the chi-square distance of the gaits to diagnose the PD, while studies^21,22^ evaluated the dynamics during freezing of gait in patients with the PD. As one of the possible non-invasive methods researchers also employ wearable sensors to record signals such as signals of electrical brain activity, i.e. electroencephalograms (EEG). The EEG signals can be used to characterize neuropsychological activity connected to the PD and are reliable method for monitoring the PD progression^23^. Numerous approaches can thus be used in order to help in detection and recognition of possible PD. To differentiate between the EEG signals of PD patients and healthy subject diverse machine learning strategies are often employed. Among those, machine learning for cognitive profiling in the PD was used in^24^. Another study^25^ used higher order spectral features to separate between the EEG signals of PD patients and healthy subjects. Oh et al.^26^ employed a convolutional neural network, while a study^27^ analyzed effects of using various neural network functions on the ability to differentiate between the EEG recordings of PD patients and healthy subjects. Another study^28^ employed the EEG channels cross-correlation, while in^29^ they employed wavelet transform for feature extraction and combined it with support vector machine and multilayerperceptron based classifier to detect the PD from EEG signals. To improve the detection of PD patients, EEG signals together with the electromyogram signals were also used in the past^30^.

The studies which differentiate between the healthy subjects and patients with the PD based on the EEG signal analysis used different features of the signals. Although these studies have achieved good differentiation between the healthy subjects and patients with the PD there is still a pressing need to identify various features and EEG channels which could help in reliable diagnosis of the PD in a patient. In previous years different studies explored viability and proposed features extracted using diverse methods and electrode placements to facilitate robust PD detection. A study^31^ explored various methods to select the optimal feature selection. Another study^32^ used 19 electrodes to detect brain activity in basal ganglia, while study^28^ used cross-correlation of EEG channels to select pairs of channels and binary classifier to select the number of channels.

In this paper we propose and investigate use of two techniques, common spatial patterns (CSP) and Laplacian mask. We employed these techniques with the aim of deriving robust features enabling separation of EEG records belonging either to a group of healthy subject or a group of patients with the PD, subjected to an auditory task. Another aim of this study was to propose and investigate the use of the CSP technique to find the most informative channels within the EEG, reducing the number of EEG signals required for the classification.

### Background

A study^33^ classified EEGs of controls and PD patients on the basis of the habituation to auditory events (*Standard*, *Novelty*, *Target*). The authors reported achieving the best accuracy when classifying the records of 82% when using around 20 features. So far this is the best accuracy achieved using this dataset. To match these results we used classical techniques for feature extraction, such as sample entropy, root mean square, median frequency and peak frequency. We divided frequency range in typical EEG bands (e.g. delta, theta, alpha, beta and gamma). Next, we calculated features for each record for different auditory task. Each feature was calculated as an average of the features extracted for each interval for a particular auditory task, yielding three numbers for each of the frequency bands for each feature for each channel of each record. This yielded over 3500 features for each record. For the classification we selected several features which seemed most promising. The best classification accuracy was achieved using 10 features, which yielded classification accuracy of 90%. Although these results seem promising, there is a problem that in all likelihood the classifier was overfitted to the data. It seems, that more informative features are required, which could help in distinguishing between the EEG records of healthy subjects and patients with the PD. We investigated two techniques and employed them in a new way with the aim of determining the most informative EEG channels and deriving small set of features which would allow for robust separation of the two groups.

## Methods

We used the publicly available EEG records, which were obtained in the scope of a study regarding the habituation to novel events^33^. For that study, the University of New Mexico Office of the Institutional Review Board approved the study. All participants also provided written informed consent. All methods were performed in accordance with relevant guidelines and regulations.

### Materials

For this study we used the EEG records, that were obtained in the scope of the study exploring differences in the habituation to auditory events between the patients with the PD and healthy controls^33^. The records and other data pertaining to the records are available through the Predict repository. The records in the database contain 60 EEG channels across 0.1 to 100 Hz frequency range sampled at 500 Hz, with additional collected information for each of the patients (e.g. age, gender). The database consists of 75 EEG records of 50 subjects (25 healthy and 25 PD patients). For each of the healthy subjects one EEG record exists in the database, while there are two EEG records for each of the PD patients. Both groups were asked to undergo the auditory oddball tasks, while the recording was taking place. Healthy subjects had to undergo these auditory oddball tasks once, while PD patients had to undergo these tasks twice, one week apart. During one of the takes of the tasks the PD patients were off their medications for the period of 15 h. One take consisted of several runs, where the subjects were exposed to different auditory sounds (designated as *Standard*, *Novelty*, *Target*), while the EEG scalp recordings were taken. There was no discernible difference in general data between the control subjects and PD patients. Altogether 9 female and 16 male patients diagnosed with the PD, and also 9 female and 16 male control subjects were included in the study. Average age of the patients with the PD was 69.98 years, while the average age of the control subjects was 69.32 years. The patients with the PD also underwent neuropsychological and questionnaire assessment.

### Data processing

Artifacts due to eye blinking were removed following the Independent Component analysis. The EEG signals were then filtered using the two-way least-squares FIR band-pass filter. Previous study^33^ indicated that the most information allowing separation between the two groups (controls and PD patients) was contained primarily in low frequency (Delta) band. Due to this reason we decided to leave the lower frequency for this study at 0.1 Hz in order to remove only very low frequencies, and only vary the upper frequency of the band-pass filter. The signals of the EEG records were then band-pass filtered using several bands: *0.1–4 Hz*, *0.1–13 Hz*, *0.1–20 Hz* and *0.1–30 Hz*. The EEG signals for each record were split into segments according to different auditory tasks (*Standard*, *Novelty*, *Target*). Each segment was 4 seconds long (2000 samples). The start of the task was centered in the middle of the given segment. From each of the segments we extracted intervals of different lengths (e.g. 1000 ms, 500 ms and 250 ms), starting at different times after the start of the auditory task to identify the most informative part of the signal.

Next, we constructed the average interval containing 60 EEG channels for each group (controls and PD patients) and each auditory task. There are two possible ways: namely as an average of all average intervals, which were calculated for the records in the group; and as an average of all intervals in the group. To construct the average interval in the first way the average of all intervals belonging to a given auditory task for each record has to be constructed. The average intervals belonging to EEG records in a given group for a given task have to be averaged to obtain the average interval comprising of 60 channels. For the average interval constructed in the second way all intervals for all records in the given group for an auditory tasks have to be averaged. To construct average signal for this study we used the second approach.

### Common spatial patterns

To obtain the optimal features of the EEG records of the two sets we employed the CSP^34^. The CSP algorithm consists of several steps.

- First, the matrix *E* of $M \times N$ size is constructed, where *M* is number of EEG channels and *N* is the interval length in samples.
- In the second step, the covariance matrix *C* for the matrix *E* is calculated by multiplying matrix *E* with its transposed version and dividing the resulting matrix with the sum of diagonal values. The above procedure is performed for the two classes of EEG signals, and the resulting covariance matrices are summed to produce the composite covariance.
- In the next step, the whitening transformation is performed. For this transformation, first the eigenvectors and eigenvalues are calculated. Then the square root of the inverse of diagonal matrix of eigenvalues is multiplied with the matrix of eigenvectors to obtain the whitening transformation *W*.
- This is then used to transform matrices *E* for both classes, to obtain whitened EEG data matrices, which share common eigenvectors. Projecting the whitened EEG data on the first and last eigenvectors yields feature vectors which are optimal in the least squares sense. The obtained matrix *W* can then be used for mapping of EEG signals into component space.

For feature extraction we had to calculate the matrix *W* for signal mapping first. Since there are several records in each group (control and PD) and each record contains numerous intervals belonging to a given auditory task, we had to calculate average interval for each auditory task (*Standard*, *Target* or *Novelty*) and for the two groups (controls and PD patients). Average signals for a specific task and for a given group were summed and divided by the number of intervals:

1 $$\begin{aligned} a_{g}(i,j) = \frac{1}{z_{tot}}\sum _{k=1}^{M}\sum _{l=1}^{N_k}s_{g,k}(i,j) \end{aligned}$$

where *g* indicates either control or PD group, *M* represents number of records of a given type (control or PD) in a set, $N_k$ represents the number of intervals of a given type (*Standard*, *Target* or *Novelty*) for a *k*-th record, *i* represents channel number, *j* represents sample number in the selected interval, while $z_{tot}$ represents total number of intervals of a given type for the entire group of records: $z_{tot} = \sum _{k=1}^{M}N_k$. Averaged signals for a given auditory task for the two groups were then used as an input into the CSP algorithm ($E_{g} = (a_{g}(i,j)), i=1..M, j=1..N$). As the output of the algorithm we obtained the mapping matrix *W*, which extremizes variances of the two groups. Since there are several intervals for each auditory task, we calculated the mapping for a given record and task in two ways. We calculated average of all intervals for given auditory task in the record, which was then mapped into component space, and we also calculated mappings for each of the intervals and then averaged mappings to obtain mapping for the record and the task. To obtain the single value feature for each signal of the given record we calculated the logarithm of the variance of the given signal in the interval of the segment. Selected features were then used for the classification of records.

Besides the original version, various versions of the CSP algorithm are commonly used in brain computer interfaces to distinguish between the EEG signals from the subjects performing two distinctive tasks^35–38^.

### Laplacian mask

As another technique to extract features we used the Laplacian mask. This is a differential operator, and is extensively used in image processing and also in signal processing. For EEG processing, the computation of Laplacian mask consists of determining the central electrode and then subtracting quarter of the values of the neighboring electrodes:

2 $$\begin{aligned} y_{(r,c)}(n)=x_{(r,c)}(n)-\frac{1}{4}(x_{(r-d,c)}(n) + x_{(r,c-d)}(n) + x_{(r+d,c)}(n) + x_{(r,c+d)}(n)), \end{aligned}$$

where *n* indicates the sample number, *r* and *c* indicate relative row and column number (assuming that scalp electrodes are mapped onto a 2-D matrix), while $d \in \{1, 2\}$ indicates neighboring channel (in this 2-D matrix, where *d* represents immediate neighbor if $d=1$, or next to immediate neighbor if $d=2$, in horizontal or vertical direction).

The mapping for a given EEG record using the CSP was performed in two ways, due to numerous intervals for each auditory task. This was not required for the Laplacian mask for a given record, since calculating the Laplacian mask of the average interval yields the same result as averaging results of Laplacian mask for each interval. For the feature extraction we first averaged intervals belonging to a given auditory task using Eq. (1) and then we used Eq. (2) to calculate values of the Laplacian mask for the entire averaged interval. Finally, all the calculated values in the interval of the segment for given auditory task were averaged to obtain a single value, which we used as a classification feature.

### Classification

For the classification we extracted set of features for each record. The features could be derived in two ways, namely the average of the intervals for the auditory task to derive the feature could be employed or the features for each of the intervals could be obtained and then averaged to obtain the feature for the record. We used the first possibility. To perform the classification and to obtain the real world performance we divided the entire set into a learning and validation set. The division was performed before the start of the study and has not changed during the study. Learning set comprised of 60% of the records (15 records were from the patients with the PD and 15 from the controls), while validation set contained the remaining 40% of the records (12 from the patients with the PD and 12 from the control subjects). We used the learning set to train the classifiers, while the validation set was used to assess the classification performance.

We used different classifiers to find the one with the best possibility of separating the groups. We tested the *Support Vector Machine* using either the polynomial kernel or the radial basis function (RBF), the *Bayes* classifier and the *Linear* and *Quadratic Discriminant Analysis* (LDA and QDA respectively) classifiers.

For the classification we used features obtained using the CSP and the features obtained using the Laplacian mask. Although the CSP is used to separate the signals belonging to different tasks performed by the same subject (e.g. motor movement imagery), we decided to try instead to use this method to separate the signals belonging to subjects in the two groups (control and PD), which were subject to the same auditory stimulus (task). In this study the *Standard* task was used. To obtain features using the CSP we used the CSP method on the learning set to obtain the mapping matrix *W*, which was next used on the training set to extract features for each of the records used for learning. The obtained matrix *W* was then used on the validation set to extract the features for each of the records, which were then used for the classification.

To classify the records using the Laplacian mask or combination of masks we calculated average intervals for a selected task for each of the records in the learning set first and than extracted classification features for the records in the learning set, which were then used to train the classifiers. Next, we used Laplacian mask or a combination of masks to extract the features for the records in the validation set, which were then used for the classification.

## Results

**Figure 1:**
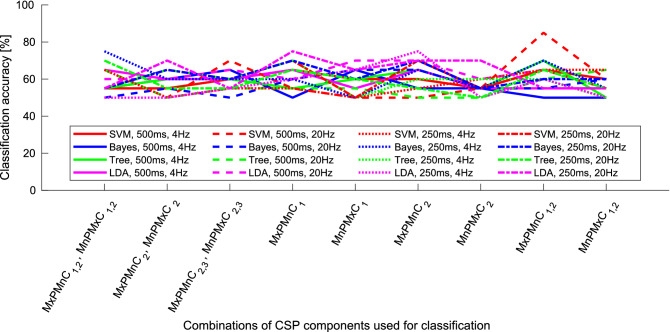
Classification results of the validation set when different parameters for feature extraction and classification were used when using the CSP. Shown are results when the upper frequency of the band-pass filter was 4 Hz or 20 Hz, the length of interval was 250 ms or 500 ms, the classifier used was either SVM$_{RBF}$, Bayes, Decision Tree or LDA, for different combinations of CSP components. On x-axes are plotted combinations of channels, where MxPMnC$_l$ denotes selected components (*l*) with maximized variance for PD group and minimized variance for control group, while MnPMxC$_{l}$ denotes selected components (*l*) with minimized variance for PD group and maximized variance for control group. For better visibility results for only selected values for upper band frequency and interval length are presented. Omitted are also the results obtained with SVM classifier with polynomial kernel and QDA classifier, since the results were similar to those obtained using the SVM$_{RBF}$ and LDA, respectively, although generally lower. Figure was created using Matlab 2020b (https://www.mathworks.com/products/matlab.html).

We used the learning set, which contained 60% of the database, to train the classifiers. These classifiers were then used on the validation set, containing remaining 40% of the database, to asses the expected performance in the real world. When using CSP usually features for first and last three components with extremized variances are calculated and used for the classification. To obtain the optimal results of classification we extracted and tested different combinations of components for feature extraction and different values of parameters. Components were selected from the first three components, which have maximized variance of the PD group and minimized variance of the control group (MxPMnC), and from the last three components, having minimized variance of the PD group and maximized variance of the control group (MnPMxC). We also tested different interval lengths, upper band frequencies of band-pass filter and classifiers. Figure 1 shows the results when using combinations of parameters used for feature extraction and classification. The best results were obtained using the SVM classifier with RBF kernel (SVM$_{RBF}$) when the upper band of the band-pass filter was set to 20 Hz, the interval length was 500 ms and only the first two features, extracted from the second and the third CSP component with maximized variance for the PD group and minimized variance for the control group (MxPMnC$_{1,2}$), were used. The classification accuracy on the validation set was 85% (sensitivity of 70% and specificity of 100%) for the *Standard* task, which improves on the classification accuracy reported in^33^. Classification results using other combinations of parameters were lower and did not exceed 75%. Generally slightly better overall results were obtained when using the 20 Hz upper band frequency of the band-pass filter and 500 ms interval for feature extraction. According to these results we can also see, that generally SVM$_{RBF}$, Bayes and LDA classifiers performed better as Decision tree classifier.

**Figure 2:**
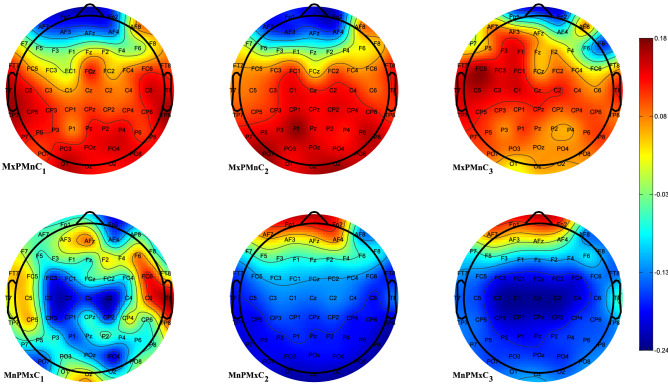
Topological plots of EEG channel contributions. In the top row are presented plots for the signals with maximized variance for the PD group and minimized variance for the control group (plots MxPMnC$_{l}, l=1,2,3$), while in the second row are plots for the signals with the minimized variance for the PD group and maximized variance for the control group (MnPMxC$_{l}, l=1,2,3$). For the calculation the interval of the length of 500 samples was used. Signals were band-pass filtered using the [0.1–20 Hz] filter. Figure was created using Matlab 2020b (https://www.mathworks.com/products/matlab.html).

Next, we investigated the ability of classifying the EEG records belonging to the two groups when using the features obtained using the Laplacian mask. First, we determined perspective channels for feature extraction. We calculated the average intervals for each of the tasks for the two groups (control and PD), which were used to obtain the matrix *W* for the two groups for different auditory tasks. We used the inverse of the first and the last three components from the obtained matrix *W* to identify the most informative channels. As the most informative electrodes we selected those electrodes, which exhibited substantial changes in close vicinity of the electrode. Figure 2 shows the obtained plots with maximized variances for PD and minimized for control groups (plots MxPMnC$_{l}$, $l=1,2,3$), and minimized variances for PD and maximized for control groups (plots MnPMxC$_{l}$, $l=1,2,3$). In Fig. 2 we see, that the values for a given channel in the extremized plots are not substantially different for *l*-th component, with maximized variance of the PD group and minimized variance of the controls, and *l*-th component with minimized variance of the PD group and maximized variance of the controls (e.g. channel F3 shows larger difference only for plots MxPMnC$_{3}$ and MnPMxC$_{3}$, but not for MxPMnC$_1$ and MnPMxC$_1$, or MxPMnC$_2$ and MnPMxC$_2$). Despite that, we expected, that the contributions of neighboring channels when calculating Laplace mask for the records belonging to the groups would further help in separating the two groups by introducing, to various degrees, information from neighboring channels. Based on topological plots we identified several channels as a possible center of the Laplacian mask, which exhibited substantial variances in their neighborhood as can be observed in Fig. 2: F3, F$_{Z}$, F4, FC3, FC$_{Z}$, FC4, C3, and CP$_{Z}$. We also included channel C4 to maintain the symmetry of the selected channels. Some channels had to be omitted due to the inability of calculating Laplacian (i.e. not having all neighbors). We also calculated features using the large Laplacian mask where possible, where we skipped the immediate neighbors in all directions and instead took channels that were further from the center (*d* has value of 2 in Eq. 2).

**Table 1:** Classification results using validation set for different combinations of values.

Acc (%)	Se (%)	Sp (%)	Int. len. (ms)	Laplacian mask	Frequency	Classifier
90.0	90.0	90.0	1000	F$_Z$, F3$_L$, F4$_L$	0.1–4,13,20,30 Hz	Bayes
90.0	80.0	100.0	1000	F$_Z$, C3$_L$	0.1–30 Hz	LDA
90.0	90.0	90.0	500	F$_Z$, F3$_L$, F4$_L$	0.1–4,13,20,30 Hz	Bayes
90.0	80.0	100.0	500	F$_Z$, C3$_L$	0.1–30 Hz	LDA
90.0	90.0	90.0	250	F$_Z$, F3$_L$, F4$_L$	0.1–4,13,20Hz	Bayes
90.0	80.0	100.0	250	F$_Z,$ C3$_L$	0.1–4 Hz	SVM$_\text {RBF}$

Shown are the best results when using interval length of 250 ms, 500 ms and 1000 ms, different Laplacian masks, upper frequency band of 4 Hz, 13 Hz, 20 Hz and 30 Hz, and several classifiers (Linear Discriminant Analysis (LDA), Quadratic Discriminant Analysis (QDA), Bayes and Support Vector Machine (SVM) with radial basis function (RBF)). Subscripted $_L$ in channel designations of Laplacian masks indicates large Laplacian mask.

**Figure 3:**
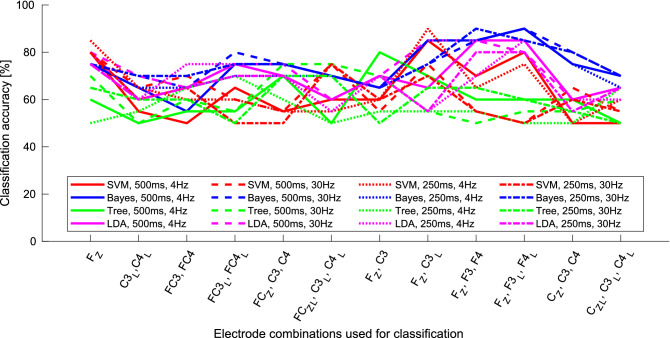
Classification results of the validation set when different parameters for feature extraction and classification were used when using the Laplacian mask. Shown are results when the upper frequency of the band-pass filter was 4 Hz or 20 Hz, the length of interval was 250 ms or 500 ms, the classifier used was either SVM$_{RBF}$, Bayes, Decision Tree or LDA, for different combinations of CSP components. On x-axes are plotted combinations of channels for positioning Laplacian mask, where *L* in subscript indicates large Laplacian mask. For better visibility results for only selected values for upper band frequency and interval length are presented. Results obtained with SVM classifier with polynomial kernel and QDA classifier are also omitted, since the results were similar to those obtained using the SVM$_{RBF}$ and LDA, respectively, although generally lower. Figure was created using Matlab 2020b (https://www.mathworks.com/products/matlab.html).

We obtained better classification results when using the features calculated with the Laplacian mask as were those obtained using the CSP. We also tested how different parameters affect classification results, e.g. Laplacian mask position, using more than one Laplacian mask, changing the upper band frequency of the band-pass filter and varying length of the interval for feature extraction. We also tested different classifier. Using combination of different masks we managed to achieve classification accuracy of 90% on the validation set with various combinations of parameters. Table 1 shows combinations of different values of parameters with which the highest classification results were obtained. The results in Table 1 show, that the best separability was achieved using combination of several Laplacian masks. We can also see from the results in the Table 1, that when using three features, the sensitivity and specificity were balanced, while when using only two, sensitivity dropped, while specificity rose. Figure 3 shows results of classification when different values of parameters were used. Similarly as before, better overall results were obtained when using the higher upper band frequency of the band-pass filter (in this case 30 Hz). Selection of interval length for feature extraction also affected classification results, where overall slightly better results were obtained with the interval of the length of 500 ms. According to these results Bayes and LDA classifiers performed generally better as Decision tree and also SVM$_{\text{ RBF }}$ classifiers. The most important role in the classification played the selection of channels for feature extraction. According to results, from the 8 selected informative channels, four are such, that in combination, offered very good separability between the groups. The most promising seems to be channel F$_{Z}$. Promising channels are also channels F3, F4 and C3, when using normal or large Laplacian mask. Several classifiers using channels F$_Z$, F3 and F4 (different combinations of parameters interval length and upper band frequency of band pass filter) as well as several classifiers using electrodes F$_Z$ and C3 (different combinations of parameters) achieved classification accuracy of 90%. Classification when using features obtained on other channels yielded lower classification accuracy, which was still at least 80% in several cases.

## Discussion

Parkinson’s disease is a neurodegenerative disease for which no cure yet exists^39^. But treatments exist^40^, which can help alleviate and control the symptoms of the disease and maintain the quality of live. Early diagnosis of the disease can thus initiate early treatment, preferably even before the motor symptoms emerge, since by that time there is already a significant neurological damage. Proper and early treatment is important since it is supposed to be more effective in early stages of the disease and can significantly delay the need for additional antiparkinsonian treatments^41^. Also non-pharmacological treatments can be performed more easily in early stages and might help in slowing the progression and improving quality of life^42^.

In this paper we investigated use of different techniques to extract features from the EEG, which could help in separating EEGs belonging to PD patients from those belonging to healthy subjects before the motor symptoms emerge. The first technique, which we investigated, was the CSP technique. This technique is mainly used to separate EEGs of a person performing two distinct tasks as a means of implementing brain–computer interface. We used this technique in a different way, namely, we tried to separate the EEGs belonging to either a healthy subject or a PD patient performing the same task. The reasoning behind using this technique in this way is that the PD affects responses to a given task. Therefore, EEG signals of a subject performing a given task should be different for a PD patient as are for a healthy subject. Consequently, this method can be employed on EEG signals belonging to PD patients and to healthy controls performing the same task. Use of this technique then allows to differentiate between EEG signals of PD patients from those of healthy controls. This allows to obtain the transformation for extracting features enabling separation between the EEG records belonging to either of the two groups. The difference is that the same task is performed by the subjects in one of the two groups instead of two tasks being performed by a given subject. To obtain the transformation the averages of signals when performing a given task has to be performed. This also makes the method more resilient to possible noises, which are attenuated, and might otherwise skew the results. The results indicate, that this technique can be used for this task, although selection of appropriate parameters is extremely important. With proper selection of parameters the classification accuracy was higher as was the classification accuracy of the authors of the database^33^, that was used for this study. Despite good classification results, results also show, that adjustments of different parameters caused drop of classification performance.

The most important parameter appeared to be the selection of components in component space. Classification using the features extracted from components with maximized variance of the PD group and minimized variance of the control group generally yielded higher accuracy as did classification using the features extracted from components with minimized variance of the PD group and maximized variance of the control group. On the other hand, despite having to select components in component space, this technique otherwise does not require selecting the most informative channels. The selection of contributions of the most informative channels is already performed by the method itself, and allows good classification with only a limited feature set.

According to results, the interval length and the upper bound frequency also affect the classification results when CSP is used for feature extraction, although the influence is not as important as is component selection. Results show, that generally better performance is obtained using the 20 Hz upper band of the band-pass filter. Use of the 500 ms interval for feature extraction also yielded generally slightly better classification results, when the CSP method was used for feature extraction.

We also investigated the use of the Laplacian masks for feature extraction, where different masks and combinations were used for classification. For the selection of the most informative channels we used the CSP transformation (the matrix *W*), to observe contributions of different channels to extremized signals. The CSP method calculates the contributions of different channels, indicating, which channel contributes the most to a given extremized component. By observing contributions of the channels and their surroundings we identified several possible candidates for feature extraction using Laplace mask. We also tested selected values for different parameters and several combinations of identified channels to extract features and perform classification. The highest classification accuracy was 90% and was even higher as when using the CSP. This accuracy was obtained using different values of parameters and different combinations of selected channels, indicating that this method is relatively robust. Results indicate that more then just one channel (feature) is recommended, and also that the channel F$_Z$ should be among selected channels for feature extraction. Use of only one feature generally did not offer enough separability between the groups of records.

We used CSP technique to identify the most informative channels for feature extraction using the Laplacian mask. Results support our initial selection of channels for feature extraction, indicating, that CSP is also promising technique, when channel selection is required. Interestingly, using the CSP to identify the most promising channels, we initially did not include channel C4. We added this channel so that the channels were selected symmetrically on both parts of the head. Classification results using also this added channel C4 were generally lower. The selection of channels for feature extraction also supports results in^33^, where the most informative features were predominantly in frontal area.

For the classification of the features extracted using Laplacian mask the best suited classifiers seem to be Bayesian classifier, followed by LDA, while Decision tree seems to be the least suited. The performance of the Bayes classifier was similar to that of the LDA but with balanced sensitivity and specificity.

Previous study^33^ showed, that low frequency content is important and that higher frequency bands contain less relevant information. Our results support their finding, that the majority of information is contained in the low frequency area, but also show, that some important information, allowing separation between the groups, is also present in higher frequency bands. Depending on the interval length and selection of classifier, higher classification results were generally obtained when using also the higher frequencies, which can be also observed in Fig. 3 (e.g. solid lines generally above dashed lines, but not always, dotted lines generally above dash-dotted lines).

Two aspects, which can influence the classification performance are also length of the interval for feature extraction and the start of that interval. We did not present the results of classification when features were extracted using different start times of intervals, since we did not observe any significant changes in classification performance. Possible reason is, that the start times were selected only after the initial response to auditory task has already diminished. Start of an interval was in each case on a more or less linear slope, which resulted in negligible overall influence on the classification performance. On the other hand, the length of the interval actually affected classification performance. With longer interval length for feature extraction the classification results were generally slightly better, which can be also observed in Fig. 3 (e.g. solid lines are generally above dotted lines, dashed lines are above dash-dotted lines). The reason might be that the signals, despite filtering, still contain some random noise, which can affect the classification. By using longer intervals for feature extraction such noises are more attenuated.

## Conclusions

Diagnosis of Parkinson’s disease needs to be set in the early stages of the disease. Waiting for motor symptoms to appear might be too late for timely treatment so other techniques are needed. Analysis of EEG signals offers inexpensive and non-invasive alternative. Numerous analysis approaches have been proposed in the past. In this paper we investigated use of the CSP method and Laplacian mask method for feature extraction to classify EEG records belonging to the groups of healthy controls and those belonging to PD patients. We have shown, that both methods can be used to extract features allowing to separate EEG records belonging to the two groups. The results show, that separation between the two groups might be possible with a relatively small set of features (two to three features) using either of the methods. We have also shown, that the CSP method can be used to perform selection of most informative channels. The results indicated that the most informative channel for classification using features extracted with Laplace is F$_Z$, while when using the CSP, the method itself performs selection of channels. The classification accuracy on the validation set using features extracted using either of the techniques was quite high. Furthermore, only a few features were required for the classification, which avoids classifiers to be over-fitted to the training data.

The problem of separating EEG records from the two groups is not an easy one. We employed two known techniques in a new way to explore the possibility of using them for this task. Both techniques proved to be capable of delivering features allowing separating EEG records of subjects belonging to different groups.

## Data Availability

The dataset supporting the conclusions of this article is available in the Predict repository, http://predict.cs.unm.edu/.
